# Undeclared (Poly)phosphates Detection in Food of Animal Origin as a Potential Tool toward Fraud Prevention

**DOI:** 10.3390/foods10071547

**Published:** 2021-07-04

**Authors:** Radmila Pavlovic, Federica Di Cesare, Francesca Longo, Franco Abballe, Sara Panseri, Rossana Claudia Bonanni, Rocco Baccelliere, Bruno Neri, Luca Maria Chiesa

**Affiliations:** 1 Department of Health, Animal Science and Food Safety, University of Milan, Via Celoria 10, 20133 Milan, Italy; radmila.pavlovic1@unimi.it (R.P.); federica.dicesare@unimi.it (F.D.C.); luca.chiesa@unimi.it (L.M.C.); 2 Laboratorio Chimica degli Alimenti, Istituto Zooprofilattico Sperimentale del Lazio e della Toscana “M. Aleandri”, Via Appia Nuova 1411, 00178 Rome, Italy; francesca.longo@izslt.it (F.L.); rossana.bonanni@izslt.it (R.C.B.); rocco.baccelliere@izslt.it (R.B.); bruno.neri@izslt.it (B.N.); 3 Thermo Fisher Scientific, Strada Rivoltana, 20090 Rodano, Italy; franco.abballe@thermofisher.com

**Keywords:** food safety, (poly)phosphates, adulteration, food, ionic chromatography, high-resolution mass spectrometry

## Abstract

(Poly)phosphates are approved as water-preserving and emulsifying agents that improve the appearance and consistency of many food products. The labelling of added (poly)phosphates is essential for protecting vulnerable population groups and to prevent unfair trade practices resulting in economic fraud. The problems with (poly)phosphates’ utilisation concerns both analytical and legislative issues, such as: (1) their straightforward detection; (2) excessive addition altering freshness perception and misleading consumers; (3) uncontrolled usage increasing foodstuff weight; (4) application in products where they are not permitted; and (5) no indication on the label. Bearing all these issues in mind, the main purpose of this study was the quantification and screening of the (poly)phosphates profile in meat, marine and dairy products (160 samples), of which 43 were without declared (poly)phosphate treatment. Analysis was completed by high-performance ion-exchange chromatography either with conductometric detection or coupled to Q-Exactive Orbitrap high-resolution mass spectrometry. Although the (poly)phosphates profiles varied greatly according to species and processing type, the following criteria for detection of illicit treatment were established: high orthophosphate level, quantified short-chain (poly)phosphate anions and the presence of long-chain forms. In conclusion, the instrumental platforms used in this study can be recommended to inspection bodies as reliable methods for the detection of food adulteration with (poly)phosphates.

## 1. Introduction

Polyphosphates are food additives belonging to the category of thickeners, stabilisers and emulsifiers that are legally added to different food products such as processed meat, cheese, dairy products, seafood, etc. [1]. (Poly)phosphates are effective in improving food product quality and extending shelf life [2]. Moreover, these additives have other functions, such as prevention of decomposition and discolouration, as well as pH buffering [3].

The term “polyphosphates” refers to a class of linear or cyclic phosphate anion condensation chains composed of residues of orthophosphate linked by means of phosphoanhydride bonds [4,5]. (Poly)phosphates are delineated in Annex II to Regulation (EC) No 1333/2008 on food additives (Commission regulation EC No 1129/2011), as amended (231/2012) [6,7]. Unfortunately, already for this first classification discrepancies and misinterpretations can be found with regard to current control procedures on foodstuffs of animal origin. Concretely, pyrophosphates and triphosphates are often mistakenly included in the polyphosphates category, which is extremely confusing as far as regulatory issues are concerned. Commission Regulation EC No. 1129/2011 assigns different identification codes to the different types of phosphate additives; E450, E451 and E452, respectively, stand for pyrophosphates, triphosphates and (poly)phosphates, indicating strict legislative distinctions [6]. Furthermore, their functions are different, and they are used for distinct food matrices. The main prerogative of E452 is retention of water in food products; hydrolysed into different orthophosphate units, it produces the desired effect of softening the texture and maintaining the elasticity and shape of the flesh. Pyrophosphates and triphosphates are limited in this function, as they consist of only a few orthophosphate units; they are therefore mainly used as melting salts. This is why the word “(poly)phosphates” is used in the rest of this report, to indicate the entire class of these additives.

Until recently, (poly)phosphates had not been recognised as toxic, but inflated consumption of phosphate resources can express the detrimental effects on the bones and cardiovascular system. Therefore, a reasonable consumption (up to 40 mg phosphate per kg of body weight per day) seemed to pose no health risk [8,9].

However, lately the health risks associated with (poly)phosphates have been brought to the fore [10,11]. Alarm was created when studies showed their ability to retain minerals (calcium, iron and magnesium) present in foods, thus preventing correct adsorption [12,13]. In particular, depriving the body of the correct daily dose of calcium can lead to a reduction in bone calcification, with babies and toddlers as the most vulnerable age group [14]. In addition, consumption of (poly)phosphates should also be reduced or avoided in women in menopause, those suffering from osteoporosis or in patients with compromised renal function [10,12]. 

Intensive use of (poly)phosphates has become a matter of concern for different food safety establishments. The European Food Safety Authority (EFSA) re-evaluated the safety of (poly)phosphates, emphasizing that these agents are shown to be of low acute oral toxicity and there is no concern with regard to their genotoxicity and carcinogenicity, but their intake via processed food has to be frequently monitored [9]. The EFSA Panel did not suggest any modifications in existing legislation regarding the Maximum Permitted Level (MPL) for condensed phosphates in different food items [6,9]. Limits set out in Regulation EC No. 1129/2011 are quite high, even in the range of g/kg of product MRLs for phosphoric acid, ortho-, pyro-, tri- and (poly)phosphates for the different categories of raw and processed food items [6].

A quite different issue is the deceptive, undeclared treatment of different fresh, raw food materials with (poly)phosphates [15]. This treatment extends the commercial life of the foodstuff, retaining water for a longer period, which frequently misinforms consumers [16,17]. The health risk for this type of product is obvious; in fact, the development of pathogenic microorganisms and/or degradation compounds (e.g., biogenic amines) might be sped up in fish and meat matrices. Therefore, the microbiological and chemical risk of ingesting such hazardous foodstuffs is very high and is to be considered of extreme importance.

From an analytical point of view, the determination of (poly)phosphates has always represented a major problem for bodies responsible for controlling food of animal origin. In fact, the reference spectrophotometric method [18] does not provide the direct determination of (poly)phosphates but determines only the so-called “relevant phosphorus”, i.e., phosphorus content that, in relation to the protein content, can be considered adequate [19]. This technique is non-specific and can lead to overestimates if other phosphorous-containing additives are present [20,21,22]. In Italy, this issue has been extensively addressed, making it possible to compare the current spectrophotometric reference method with those introduced recently (e.g., high-performance ion-exchange chromatography with suppressed conductometry, HPIEC–SCD) [19]. 

Hence, a requirement for more precise analytical methods for (poly)phosphate quantification was clearly supported by the EFSA Scientific Committee report [9]. Several procedures adequate to reveal the presence of (poly)phosphates in different food matrices have been established [21,22,23,24,25]. A method based on Ion Chromatography (IC) with suppressed conductometric detection (SCD) was developed and validated for the separation and direct determination of (poly)phosphates in different animal foodstuffs [25]. IC remains a predominant alternative because it was proven to be highly sensitive and able to separate the main short-chain phosphate species [21,22,23,24,25]. Nevertheless, the most important drawback regarding the HPIEC–SCD method is the direct determination of (poly)phosphate species that contain more than three orthophosphate units. The HPIEC–SCD identification of such species became troublesome as (poly)phosphates tend to hydrolyse in basic orthophosphate units, especially when the food matrix maintains a high phosphatase activity. Effectively, the low quantity of higher (poly)phosphate forms present in the real sample hinders HPIEC–SCD characterisation.

The lack of specificity and sensibility regarding the screening of higher (poly)phosphate forms has been resolved recently by this research group, involving high-resolution mass spectrometry (HRMS) in the characterisation of the (poly)phosphate profile disclosed in different kinds of seafood [26]. The presence of (poly)phosphate residues was detected in various types of fishery products by exploiting an in-house-developed HPIEC–SCD–HRMS platform, as well as to prove the feasibility of this approach as a sustenance tool for inspections in the fishery sector.

The aim of this research was to evaluate the feasibility of using HPIEC–SCD and HPIEC–HRMS for the analysis of undeclared samples belonging to different food categories (meat, dairy, and fisheries). The samples with no indication on the label regarding any (poly)phosphate treatment were randomly picked from the market and received from authorised bodies. In particular, this trial aimed to provide greater insight regarding the following issues: (1) whether the foodstuffs were treated with (poly)phosphates or not, (2) whether it is possible to identify processing with different (poly)phosphate additives, (3) set-up of unambiguous HRMS characterisation of higher (poly)phosphates species that would implicitly point toward (poly)phosphates (E452) treatment, (4) evaluation of the potential of HPIEC–SCD to be used as a screening method to support food safety controls involved in monitoring plans and (5) surveys of the values of endogenously occurring orthophosphates in relation to those eventually formed by the hydrolysis of higher additive forms.

## 2. Materials and Methods

### 2.1. Chemicals and Reference Substances

Analytical reference standard of orthophosphate as sodium phosphate dibasic dihydrate (>98% purity), pyrophosphate as sodium pyrophosphate decahydrate (>99%), triphosphate as sodium triphosphate pentabasic (>98%), trimetaphosphate as trisodium trimetaphosphate (>95%) and sodium hexametaphosphate (SHMP) (purity of 67.9% expressed as P_2_O_5_) were purchased from Sigma-Aldrich (Saint Louis, MO, USA). Sodium hydroxide pellets EMPLURA were purchased from Sigma-Aldrich (Saint Louis, MO, USA). Ultrapure water with a specific resistance of 18.2 mΩ from a Milli–RO/Milliq system (Millipore, Bedford, MA, USA) was used to prepare standard solutions and mobile phase and to dilute samples. Minisart fiberglass syringe filters (0.7 µm pore size, 28 mm diameter) were purchased from Sartorius (Gottingen, Germany).

### 2.2. Preparation of Stock and Working Solution

Orthophosphate, pyrophosphate, triphosphate, trimetaphosphate and SHMP stock solution were prepared by dissolving the appropriate weight of solid reference standard in ultrapure water with 100 µL 0.1 M NaOH added in order to obtain the concentration of 1000 mg L^−1^ expressed as P_2_O_5_, for the HPIEC–SCD, while for HPIEC–HRMS it was calculated on the basis of the nominal phosphate mass concentrations. Working solutions were prepared by dilution of standard solutions to concentrations of 10–100 µg mL^−1^ of P_2_O_5_ for the HPIEC–SCD and 0.1–10 µg mL^−1^ as the nominal concentration for HPIEC–HRMS Q-Exactive Orbitrap procedure. Both standard and working solutions were prepared daily.

### 2.3. Sample Collection

Samples were from official controls, coming from the National Additives Control Plan implemented by Lazio, Tuscany and Sicily Regions in accordance with Regulation (EU) No 625/2017 [27]. From January 2019 to April 2020, 160 samples (74 fish, 40 meat and 46 dairy products) were analysed. Fishery products included both fresh and frozen fish, processed fish and precooked shellfish, dairy products included processed cheese, soft cheese, stretched-curd cheese and desserts and meat products consisted of fresh meat, canned meat, smoked meat and meat preparations. They were from several commercial brands, present in the different Italian markets. All samples were frozen at arrival.

Due to the easy (poly)phosphates degradation, standard solutions, spiked and real samples were processed in a short period of time from preparation/defrosting (maximum 4 h) with special attention at 4 °C until injection [28].

### 2.4. Sample Preparation

Regarding the HPIEC–SCD method, sample preparation was performed according to the internal house-accredited method of Istituto Zooprofilattico Sperimentale del Lazio e della Toscana “M. Aleandri”, Rome (IT). Briefly, 2 g of previously homogenised sample was weighed in a 50 mL centrifuge tube and 100 µL 0.1 M NaOH and ultrapure water was added up to 40 mL of volume; the samples were vortexed for 1 min and then placed for 10 min in a horizontal shaker. After centrifugation for 10 min at 4000 RPM at 4 °C, 5 mL of the supernatant was purified on a fiberglass filter, discarding the first 2 mL with no further clean-up step required, and 25 μL was injected in the ion chromatograph.

Sample preparation for HPIEC–HRMS Q-Exactive Orbitrap procedure was performed according to our recently published method [26] and was the same as this for the HPIEC–SCD. The only differences regarded the addition of 10 µL of internal standard (H^[15]^NO_3_, 100 µg/mL) to the initial sample and filtration of the supernatant (~2 mL) by Anotop 10-LC filters (0.2 μm, 10 mm, Whatman), followed by dilution (1:10) with DI prior to chromatographic analysis.

### 2.5. Instrumental Conditions for Both HPIEC Methods

Both analytical procedures used an ICS-5000+ system (Thermofisher, Sunnyvale, CA, USA), consisting of a gradient pump, conductivity detector, eluent generator module equipped with KOH cartridge and AS autosampler. A Thermo Scientific Dionex IonPac AS11-4 µm (2 × 250 mm, 4 µm particle size) column with a guard column, Dionex IonPac AS1 µm (2 × 50 mm), served as an ion exchange resin, held at a temperature of 30 °C for HPIEC–HRMS method, while for the HPIEC–SCD the AS16-4 µm was chosen.

#### 2.5.1. HPIEC–SCD Specific Parameters 

Elution was carried out at a flow rate of 1 mL min^−1^, working in concentration multistep gradient mode, programmed as follows: 2 min at 40 mM, from 40 to 60 mM in 3 min and from 60 to 100 mM in 23 min, and the system was then re-equilibrated for 7 min at 40 mM. A Dionex AERS 500 4 mm anion self-regenerating suppressor was used, set at 248 mA SRS current and operating in the external water mode. Thermo Scientific Dionex Chromeleon 7 Chromatography Data System (CDS) vers. 7.2 SR4 software was used for data processing. 

#### 2.5.2. HPIEC–HRMS Q-Exactive Orbitrap Specific Parameters 

The instrumental method was the same as in a previous study [26]. The eluent flow rate was 0.30 mL/min with an appropriate gradient from 40 to 100 mM KOH in 30 min of a chromatographical run. The KOH eluent was neutralised using a Dionex anion self-regenerating suppressor set to 68 mA (ASRS II, 4 mm).

Thermo Q-Exactive Orbitrap™ (Thermo Scientific, San Jose, CA, USA), equipped with heated electrospray ionisation (HESI) source, was used for detection of different polyphosphate species. The operative parameters with negative mod acquisition were as follows: capillary temperature 330 °C, vaporiser temperature 280 °C, the electrospray voltage 3.50 kV, heath gas arbitrary units and auxiliary gas 15 arbitrary units and S lens RF level 60. 

The Xcalibur^TM^ 3.0 software (Thermo Fisher Scientific, San Jose, CA, USA) was used to control the HRMS system and the exact mass of the compounds, and to record and elaborate data. The full scan ((FS), acquisition with resolution of 70,000) was combined with a data-independent acquisition (DIA) strategy (resolution 35,000), that delivered the fragmentation spectra of (poly)phosphates anions. The scan range was from 50 to 750 *m*/*z*, while automatic gain control (AGC) was set at 1E^6^ for FS, while for DIA it was 2E^5^. The maximum injection time was 100 ms for FS, while for the DIA segment it was auto-regulated. The isolation window for precursors was 1 *m*/*z*, whereas the fragmentation of the precursors was accomplished by 10 eV collision energy. Detection of the analytes was based on the retention time (RT) of the target compounds, and on the calculated exact mass of the deprotonated molecular ions, and at least one specific and typical fragment or isotopic pattern. The formulas of the compounds, with the exact theoretical mass of the parents and the diagnostic transition, used to confirm the various (poly)phosphates species, are reported in Table 1.

### 2.6. Method Validation

#### 2.6.1. HPIEC–SCD Qualitative Method Validation

As there is no specific reference standard regarding the validation of analytical methods for food additives, in this study the method was validated by an in-house validation model, in agreement with ISO/IEC 17025, and Commission Directive 2002/657/EC or guidelines for the validation of screening methods for residues of veterinary medicines which describe the analytical parameters to be assured to verify method reliability [29,30,31,32]. For a qualitative purpose, selectivity/specificity and limit of detection (LOD) were evaluated. In addition, linearity was assessed in order to test instrumental calibration as required by the Italian accreditation body, Accredia.

- *Selectivity/Specificity*—In order to verify the specificity parameter, at least 10 blanks (samples in which the analyte is absent) for each matrix (fish, dairy and meat products) were analysed. Fresh cod, salted and dried cod, cuttlefish, squids, prawns, tuna, sardines, cobia, anchovies, mussels, lobster and hake were considered for fish matrices; and mozzarella cheese, feta, stracchino, ricotta and different brands of cream cheese, processed cheese and ripened cheese were selected as dairy products. Lastly, blanks chosen for meat products were cooked ham, gluten-free cooked ham, chicken wurstel, sausage mince, mortadella, pork wurstel, ground beef, ground pork and raw ham. Samples were commercial products without added (poly)phosphates declared on the label.
- *LOD* was evaluated by analysing the same blank samples fortified by addition of 400 µL of the 1000 µg mL^−1^ pyrophosphate, triphosphate, trimetaphosphate and sodium hexametaphosphate standard solution to 2 g of the homogenised material to obtain a concentration of 200 µg g^−1.^
- *Linearity*—Calibration curves were assessed using the following orthophosphate, pyrophosphate, triphosphate, trimetaphosphate and SHMP working solution: 10, 25, 50, 75 and 100 µg mL^−1^ (expressed as P_2_O_5_). To control the linear range of the IC method, the relationship between the area response of injected standard solutions with the corresponding analyte concentration was measured. In particular, for SHMP the area was calculated as the sum of the areas of the first ten peaks of reference solutions.

#### 2.6.2. HPIEC–HRMS Q-Exactive Orbitrap Validation

Since the analytical methods and results of this study are based on the results of our recently published research [26], the verification parameters remained the same. Method validation was extended to the three different matrices (fresh meat, fresh fish and UHT milk) regarding recovery, precision, limit of detection (LOD) and limit of quantification (LOQ). Coefficient of variability (CV) was used for intra-day and inter-day repeatability evaluation, while recovery (expressed as %) was defined by comparing the response obtained for the same blank sample spiked before and after extraction. The calculation of the LOD = 3.3 SD/b and the LOQ = 10 SD/b was used for method limits evaluation, with SD as the standard deviation of the intercept and b as the slope of the low-concentration calibration curves acquired for all three food materials.

## 3. Results and Discussion

According to international quality regulations, validation shall be as extensive as necessary to meet the needs of the given application [29,33,34,35,36,37]. The extent of the validation depends on the aim of the analytical method and the first step is to decide which performance parameters have to be considered. That is why for the two methods developed and applied in this study the validation procedures are different. Two analytical approaches were required: (1) a HPIEC–SCD qualitative method which allowed the identification of (poly)phosphates added (E450, E451 and E452) and (2) a HPIEC–HRMS quantification method that provided confirmation that the additive was not used fraudulently.

### 3.1. HPIEC–SCD Validation as a Screening Method

**Figure 1:**
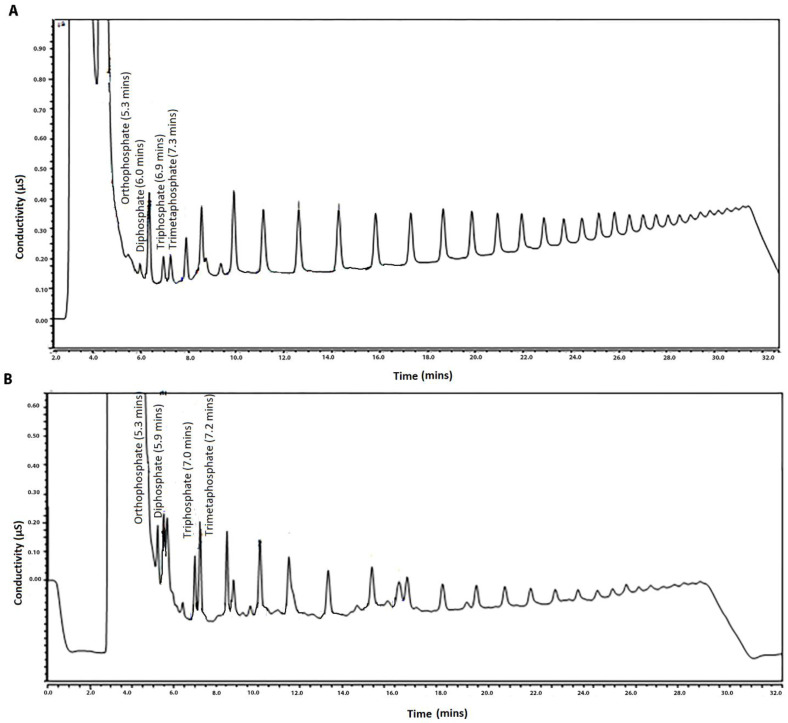
Chromatogram of a fish sample spiked with SHMP at level of 200 µg g^−1^ performed with (**A**) AS16 column and (**B**) AS11-HC column.

During the development of the HPIEC–SCD method, two different anion-exchange columns were tested in order to determine the most suitable column for the purpose of the study. Comparison was made through the chromatographic separation of a sodium hexametaphosphate standard with Ion Pac^®^ AS11-HC and Ion Pac^®^ AS16 columns. The AS16 column is of more recent production than AS 11-HC and has a greater capacity and selectivity for polyvalent anions, allowing easier elution of larger (poly)phosphates [28]. In fact, this column was more suitable for this study because it provided a larger quantity of SHMP peaks in the same time of analysis and gave a better chromatographic resolution of orthophosphate, pyrophosphate, triphosphate and trimetaphosphate signals. Lastly, the use of Ion Pac AS16 allows an appropriate separation of possible interferents in fortified and in real samples (Figure 1A,B), showing the separation of a spiked fish sample with interferents performed with an AS16 column and AS11-HC column, respectively. Those results are in line with recent findings published by Kim et al. [25] where the columns were tested as well but applied only to the seafood matrix.

**Figure 2:**
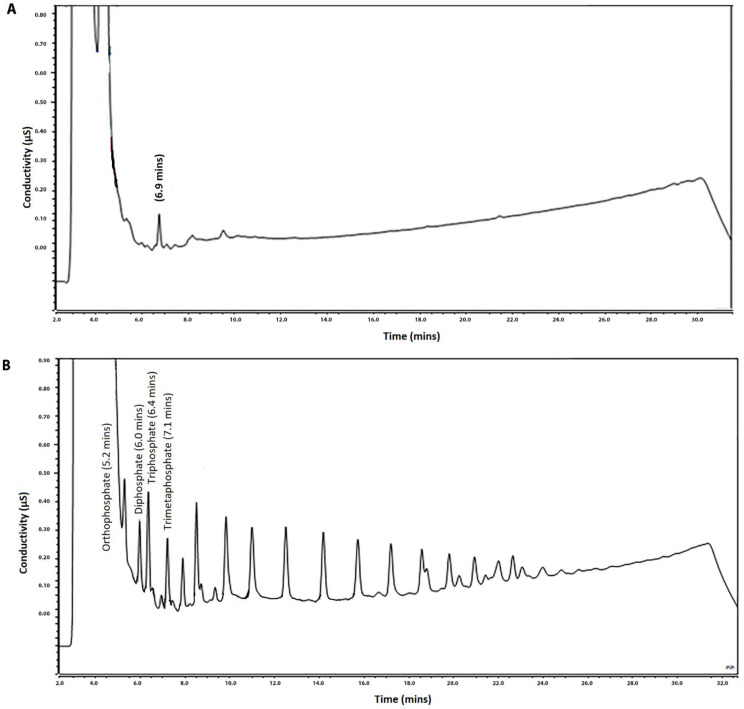
(**A**) Chromatogram of a blank dairy sample without any (poly)phosphate species detected. (**B**) Chromatogram of a blank dairy sample with SHMP added at LOD level (200 µg g^−1^).

- *Specificity/Selectivity*—12 blanks for fish products, 10 for dairy products and 12 for meat products were analysed and injected twice. In some products, e.g., plaice, stracchino and feta cheese, the presence of a matrix peak at about 6.95 min was detected, but this signal was clearly separated from peaks of pyrophosphate and trimetaphosphate. No endogenous or extraneous peak interfering with expected retention time of SHMP peaks was observed. For example, the chromatogram of a blank sample of dairy product (cream cheese) is reported in Figure 2A.
- *Limit of Detection (LOD)* of (poly)phosphates expressed as a P_2_O_5_ equivalents (as current legislation EU 1129/2011 requests) was set up at 200 µg g^−1^. All the blank samples spiked at 200 µg g^−1^, analysed in duplicate, were confirmative (S/N ≥ 3), except two (chicken wurstel and ripened cheese), which presented the interfering signals. To assess the presence of false positives, it was verified that less than 5% of the fortified samples had signals lower than the blanks’ average signal +3 sd; only one sample was (processed cheese) in the false-positive range and was then discarded. The cut-off value of 200 µg g^−1^ was fit for purpose, because (poly)phosphates are used at high concentrations in order to perform their technological utilities. Moreover, the existing confirmatory methods are unable to detect them at lower levels of concentration (e.g., 200 µg g^−1^) [18,34,35,36]. For example, a chromatogram of a LOD-spiked sample of cream cheese is reported in Figure 2B.
- *Linearity*—Calibration curves for orthophosphate, pyrophosphate, triphosphate, trimetaphosphate and SHMP, considering SHMP area as the sum of the first ten peaks’ areas, were found to be linear, with R^2^ > 0.999. The linearity test was performed on three working sessions by injecting five standard solutions for each analyte at concentrations of 10, 25, 50, 75 and 100 µg mL^−1^.

#### HPIEC–SCD Screening Method Application

The validated and accredited method was applied to samples from official controls, from the National Additives Control Plan implemented by Lazio, Tuscany and Sicily Regions in accordance with Regulation (EU) No 625/2017 [27].

Seven samples were positive to the screening method and were analysed by ISO confirmatory methods [34,35,36], and the results were confirmed for all samples.

**Figure 3:**
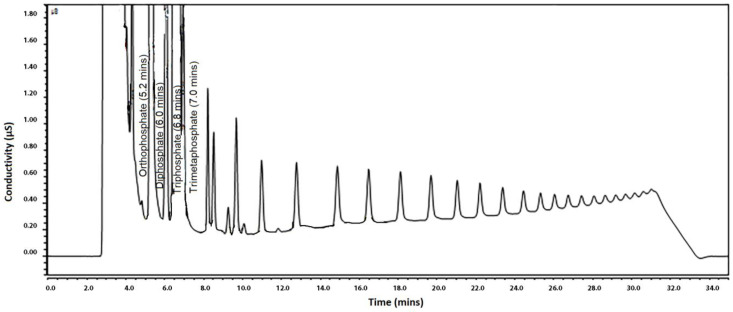
Chromatogram of a frozen shrimp sample with (poly)phosphates declared on label.

To assess applicability, commercial samples with (poly)phosphates labelled were analysed during method development. Most of the chromatograms showed only pyrophosphate, triphosphate and trimetaphosphate peaks, while the typical chromatographic profile of SHMP was present only in a few samples, as shown in Figure 3. It was therefore considered that the identification of up to the first 10 peaks of SHMP is sufficient to determine the presence of added (poly)phosphates. To confirm that peaks in real samples are due to the presence of added (poly)phosphates, the relative retention time considered as the ratio of the retention time of the pyrophosphate, triphosphate, trimetaphosphate and the additional peaks of SHMP to that of orthophosphate was calculated. Then, relative retention time for each peak of the unknown sample was compared to the ones calculated for the spiked samples at LOD level, and the ratio could not have a variation higher than 2.5%.

The identification of the (poly)phosphates peaks possibly present in the sample was made by comparing the relative retention times with those of orthophosphate. Afterwards, a further comparison was made between relative peak retention times possibly present in the unknown sample and those of the spiked sample, considering as acceptable a variance of ±2.5%.

### 3.2. HPIEC–HRMS Q-Exactive Orbitrap Method Validation and (Poly)phosphate Characterisation

Given the fact that there are no specific protocols aimed at validating the analytical procedure for (poly)phosphate determination in foods, the HPIEC method with HRMS Q-Exactive Orbitrap detection was validated by an in-house validation model, in accordance with Decision 657/2002/EC and Regulation 882/2004/EC, which describe the analytical parameters to be assured to verify method reliability [38,39,40]. The validation parameters for orthophosphate (certificated standard) and the commercially available standards (pyro-/tri-/trimeta-) are summarised in Table 2. It is evident that the HPIEC–HRMS method showed excellent accuracy, with acceptable CVs for intra- and inter-day precision indicators for all three food matrices. LOD and LOQ achieved by HRMS detection were much lower than those reported in the literature for SCD [21,22,23,24]. Additionally, the HPIEC–HRMS used in this study showed very good recovery results, which were similar for all the food matrices validated.

**Figure 4:**
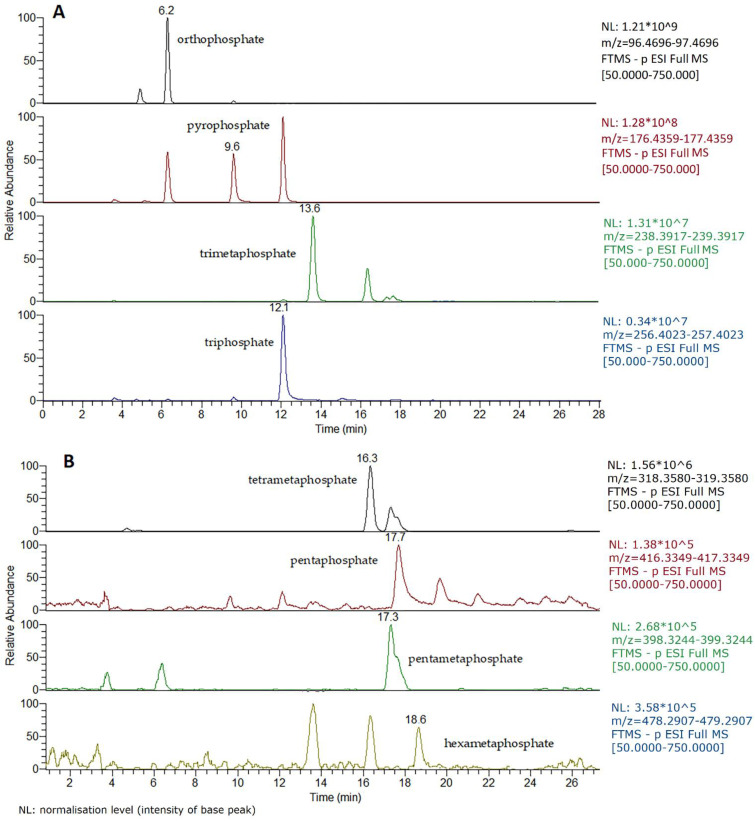
HPIEC–HRMS Q-Exactive Orbitrap ion-extracted chromatogram (TIC) of a cheese sample with declared (poly)phosphate treatment. (**A**) TIC of quantified anions: ortho-, pyro-, trimeta- and triphosphates: 2710, 5626, 6897 and 210 µg/g, respectively. (**B**) Fingerprint of the long-chain polyanions.

Despite satisfactory results of HPIEC–SCD analysis reported recently [19,25], demand for more specific methods with satisfactory robustness has arisen due to the well-known (poly)phosphate dependence on the sample matrix type. Nevertheless, HPIEC with SCD remains a priority choice for the analysis of short-chain phosphate anions, as it was proven to be very sensitive and precise for the ortho-, pyro-, trimeta- and triphosphate, although its main drawback is that it cannot identify polymeric structures with more than four orthophosphate units [19,24,25]. However, this problem has been successfully resolved with the HPIEC–SCD protocol described above [26]. This analytical HPIEC–HRMS platform showed extremely specific profiling accompanied by high selectivity, as can be seen from the ion-extracted chromatogram achieved for a cheese sample with declared (poly)phosphates treatment (Figure 4). As it was discovered by Kaufman et al. [41], during our HRMS profiling the interconversion of some (poly)phosphate anions was also observed, most presumably due to thermal impact in the ESI source. This is evident for ion 176.93595 (pyrophosphate) that appears also at the retention time of orthophosphate and triphosphate, respectively. However, such transformations do not truly affect quantification, because of the good chromatographic separation of the phosphate species.

As can be noted, the short-chain phosphate anions can be quantified utilising the calibration curves performed with their analytical standards. Unfortunately, this is not the case with higher polymeric chains which are not commercially available and thus not individually quantifiable. That is why our recently published screening (fingerprinting) approach for (poly)phosphate evaluation [26] was applied: the HRMS signals that appear in the real samples were compared with those obtained from the variable mixture of polymeric linear and cyclic (poly)phosphates, known as sodium hexametaphosphate, supplied by Sigma-Aldrich. For the full identification of main (poly)phosphate anions, the following criteria must be fulfilled: retention time, exact mass of precursor anion extracted from FS acquisition mode and MS/MS fragmentation pattern obtained in DIA analysis. The results gained herein for the declared samples confirm the most important finding published earlier by the same researchers [26]: HRMS characterisation of tetra-, tetrameta-, penta-, pentameta-, hexa- and hexametapolyphosphate anions could be used as a “confirmation fingerprint” for treatment with (poly)phosphates (additive E452).

Current legislation [6,42] specifies maximal residual levels (MRLs) expressed as P_2_O_5_ equivalents due to fact that the official AOAC method is still based on spectrophotometric measurement that does not distinguish between various (poly)phosphates but only refers to their total amount, valued as µg/g of P_2_O_5_. Thus, with regard to the high performance of the HPIEC–HRMS or HPIEC–SCD methods published earlier [18,21,22,24,25,43,44], it is indispensable to express analytical results in terms of the individual phosphate species, as has already been recommended by the European Food Safety Authority [9]. Furthermore, the ability of the method based on ion chromatography to individually quantify certain (poly)phosphates must be widely accepted by authorised bodies, as it is important to keep quantities under control: some (poly)phosphates might be harmful if consumed excessively on an ongoing basis, while there is no cause for concern if they are encountered occasionally and in low doses [45].

### 3.3. Detection of (Poly)phosphates in Real Non-Declared Samples

Various (poly)phosphates are permitted as additives for moisture control in the processing of different kinds of food materials. Their correct utilisation provides some advantages preventing biochemical and physical changes in various processed food categories [1,46] and has been given a legal framework by Regulation EC No. 1129/2011 [6]. Despite the excellent benefits, the abusive use of these additives may lead to economic fraud and has been performed by some companies with the objective of increasing product weight and obtaining greater profits [47]. However, consumers’ growing concern with regard to compositional aspects and their legitimate perplexity towards components with pure chemical origin has led to an increasing demand for “chem-free” clean labels [48]. Regulation 1169/2011 provides for transparent labelling and improves consumer protection against common fraud [6]. In order to verify conformity with legislation and to comply with correct labelling, both producers and inspection authorities have to (1) know the natural composition of food material and if there is any overlap with exogenously added substances, and (2) adopt more specific analytical techniques, e.g., IC with different detection modes [19,26]. Hence, this study analysed the undeclared food samples of animal origin belonging to the three main categories.

#### 3.3.1. Meat and Meat Products

The undeclared meat and meat product samples examined in this study were quite distinctive regarding the (poly)phosphates revealed (Table 3). Non-processed fresh meat did not contain any measurable amount of (poly)phosphates, apart from natural orthophosphate. The trace level of short-chain forms most probably belongs to the class of endogenous (poly)phosphates that has been identified in pro- and eukaryotic organisms, where they carry out an astonishing array of different functions [49]. Many bacteria, including numerous pathogens, encode the enzyme (poly)phosphate kinase that produces a certain steady-state amount of (poly)phosphates in the bacterial cytosol [50]. Therefore, bacteria can contribute to the trace occurrence of the phosphate polyanions in some fresh and processed meat samples (e.g., chicken muscles, baked turkey muscle II or cooked meat) (Table 3). Additionally, four mortadella samples contained at least one quantitatively defined short-chain phosphate, and some traces. Having a relatively low and uniform orthophosphate level, it is quite likely that those samples were not treated with (poly)phosphate additives. On the contrary, trimetaphosphate detected in the cooked ham (Table 3) points toward the utilisation of its salt as a principal additive. This form could be transformed in its linear equivalent, as evidenced by triphosphate anion occurrence.

The presence of some (poly)phosphate forms in the baked turkey muscle II and pre-cooked tripe matrix suggests that some form of E452 was applied. Furthermore, the HPIEC–HRMS detection and quantification strategy revealed unambiguous (poly)phosphate treatment in all the wurstel samples, although with completely different (poly)phosphate profiling. In particular, the high amount of orthophosphate in wurstels II and III specifies that, apart from its endogenous presence, it can be formed by the hydrolysis of phosphate additives [51]. Orthophosphates (E338) are commonly used for the adjusting and buffering of pH values; however, on their own they have a small effect on muscular protein [2]. Thus, it is not likely that E338 is applied alone in meat products and a certain percentage of its total amount can undoubtedly be attributed to hydrolysis of higher polymeric forms [2]. Therefore, the level of orthophosphate has to be taken into consideration for complete (poly)phosphate profiling.

#### 3.3.2. Fish and Seafood Products

**Figure 5:**
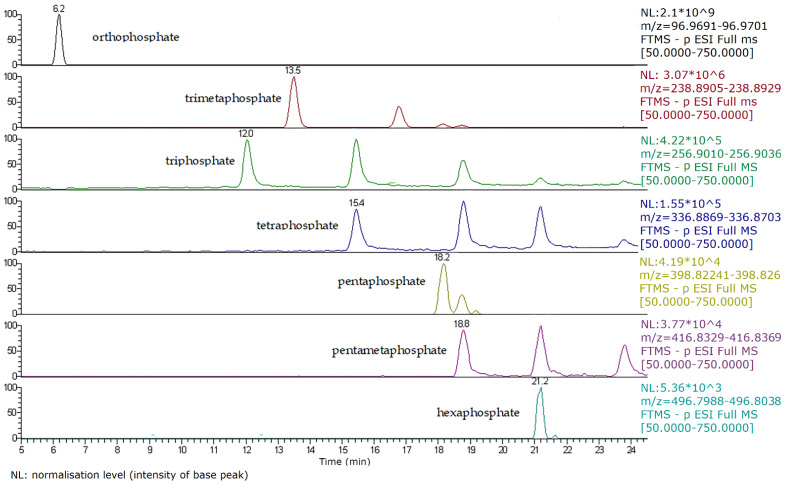
Q-Exactive Orbitrap HRMS total ion chromatogram of caramote prawn I with different (poly)phosphate anions detected. NL: normalisation level (intensity of base peak).

Regarding fish and fish products, all samples sporadically presented very low levels of short-chain (poly)phosphate species, without any higher forms detected (Table 4). The general considerations expressed above for meat samples regarding trace amounts of (poly)phosphates could also be applied for fish and marine products. Furthermore, it can be speculated that added (poly)phosphates were subjected to degradation by the intensive hydrolysis reaction catalysed by the phosphatase present in fish muscle [52]. However, in that case the concentration of monomeric orthophosphate units would be much higher than those found [25]. Therefore, the near-trace amounts detected cannot alone be used for the confirmation of illicit (poly)phosphates treatment due to fact that the concentration of orthophosphate corresponds to the naturally occurring anion [26]. For the processed marine food products, a shelled mussel sample contained pyro- and triphosphate that were accompanied by a pentaphosphate signal in the fingerprint qualitative analysis (Table 4). This clearly demonstrates (poly)phosphates application in the processing of the mussels, most probably to facilitate shell removal [17,53]. Three caramote prawn samples require special attention considering that (1) a high orthophosphate amount was detected, (2) both trimeta- and triphosphate species were quantified and (3) four characteristic (poly)phosphate species were defined in the screening evaluation (Figure 5). This three-step evaluation unquestionably proved (poly)phosphates utilisation during the processing of these seafood samples [54]. The high specificity and sensibility of the HPIEC–HRMS method used in this study is especially important for this food category, as Iammarino et al. [19] revealed the discrepancies between the two most exploited methods (indirect photometry and ion chromatography) particularly for seafood materials. These authors also emphasised two variables that could compromise (poly)phosphate detection in seafood samples: initial additive(s) quantity and storage time before analysis.

#### 3.3.3. Dairy Products

(Poly)phosphates are extensively used as an emulsifying salt in processed dairy due to their ability to sequester calcium ions and to adjust pH [55]. The complexation with calcium causes casein dissociation, increasing its solubility and hydration. Upon heating, the dissociated casein emulsifies the fat, favouring the formation of a homogeneous structure [56]. Usually, the commercial emulsifying salts are mixtures of long- and short-chain-length (poly)phosphates to ensure a balance between the chelating action and the buffering effect. In one undeclared UHT milk sample, quantification of pyrophosphate and low trimetaphosphate levels point towards (poly)phosphate application (Table 5). This finding is supported by the fact that pyrophosphate is a principal (poly)phosphates degradation product in milk [51]. Four butter samples did not exhibit any measurable (poly)phosphate species, while one of three cheese samples was particularly rich in orthophosphate, followed by a moderate triphosphate amount and complete (poly)phosphates fingerprinting. This finding once again confirmed that orthophosphate evaluation cannot be excluded from comprehensive (poly)phosphate analysis and that simultaneously performed quantification of individual phosphate species accompanied with (poly)phosphates HRMS characterisation is the best solution for detecting undeclared (poly)phosphate application.

## 4. Conclusions

A rapid and simple HPIEC–SCD method for the determination of (poly)phosphate additives in products of animal origins was developed and validated as a feasible tool for screening purposes. The method was used to detect the presence of (poly)phosphates expressed as a P_2_O_5_ equivalent at the level of interest of 200 µg g^−1^, allowing the direct identification of (poly)phosphates. It is suitable to analyse a large number of products for potential non-compliant results and to avoid false-positive samples. The positive results obtained by HPIEC–SCD were further processed with confirmatory HPIEC–HRMS. This analytical procedure was also successfully involved and used in wide-range quantification for profiling (poly)phosphate anions in the different food items to obtain more comprehensive information about additive presence when at the trace level. This enabled the detection of undeclared (poly)phosphates addition in some of the samples analysed. Consequently, this instrumental platform can be proposed to authorised bodies as a validated method to support the detection of illegal food adulteration with (poly)phosphates. The analytical strategies developed have been proven as complementary, especially when the revelation of undeclared (poly)phosphate use has to be confirmed.

## Figures and Tables

**Table 1:** Exact HPIEC–HRMS Q-Exactive Orbitrap mass database of the (poly)phosphate anions studied: elemental compositions, retention time (Rt), precursor and confirmation fragments of the parent pseudomolecular anions.

(Poly)phosphate Anion	Neutral Form Formula	R_t_ (Min)	Precursor in Full Scan (M-H)^−1^	Conformation Ions (DIA Segment)
Orthophosphate	H_3_PO_4_	5.9	96.9696	78.9590
Pyrophosphate	H_4_P_2_O_7_	9.0	176.9359	78.9561; 96.9697
Triphosphate	H_5_P_3_O_10_	11.8	256.9023	96.9697; 176.9685
Trimetaphosphate	H_3_P_3_O_9_	13.5	238.8917	118.9422; 158,9253
Tetraphosphate	H_6_P_4_O_13_	15.6	336.8686	78.9591; 256.9025
Tetrametaphosphate	H_4_P_4_O_12_	16.8	318.8580	158.9254; 256.9025
Pentaphosphate	H_7_P_5_O_16_	17.9	416.8349	256.9025; 336.8689
Pentametaphosphate	H_5_P_5_O_15_	18.9	398.8244	198.9086; 318.8582
Hexametaphosphate	H_6_P_6_O_18_	18.7	478.7907	238.8919; 318.8582
Hexaphosphate	H_8_P_6_O_19_	21.7	496.8013	247.8970; 336.8689
Internal Standard	H^[15]^NO_3_	7.0	62.9854	/

**Table 2:** Analytical performance and validation parameters of the proposed HPIEC–HRMS Q-Exactive methods for three food matrices.

Phosphate Species	Food Matrix	LOD	LOQ	Recovery (*n* = 3, Mean ± SD)			Precision (CV; *n* = 3)	
				Spiked Level (µg g^−1^)				
		(µg g^−1^)		0.1	0.5	1	Intra-Day	Inter-Day
Orthophosphate	meat	0.05	0.14	83 ± 3	88 ± 8	102 ± 7	14.6	8.2
	fish	0.06	0.18	90 ± 3	102 ± 3	96 ± 3	12.3	12.2
	milk	0.05	0.15	108 ± 3	101 ± 3	109 ± 3	9.8	4.4
Pyrophosphate	meat	0.03	0.10	88 ± 3	83 ± 3	82 ± 3	12.3	7.8
	fish	0.04	0.12	95 ± 3	91 ± 3	108 ± 3	14.2	9.2
	milk	0.03	0.10	104 ± 3	109 ± 3	104 ± 3	11.5	8.2
Trimetaphosphate	meat	0.03	0.09	80 ± 3	92 ± 3	84 ± 3	15.2	10.3
	fish	0.04	0.11	89 ± 3	92 ± 3	90 ± 3	3.5	2.1
	milk	0.03	0.10	91 ± 3	107 ± 3	90 ± 3	10.2	6.4
Triphosphate	meat	0.02	0.05	102 ± 4	107 ± 7	105 ± 3	6.2	4.3
	fish	0.02	0.06	112 ± 5	105 ± 9	110 ± 11	13.5	12.1
	milk	0.03	0.08	90 ± 8	89 ± 10	101 ± 8	12.2	9.4

**Table 3:** (Poly)phosphates in different types of undeclared meat/meat products.

Quantification in HPIEC–HRMS						Screening in HPIEC–SCD
Sample Type	Ortho-	Pyro-	Trimeta-	Tri-	Polyphosphates HPIEC–HRMS ^a^	
	(Mean ± SD; *n* = 2; µg g^−1^)					
Fresh meat						
Minced beef	781 ± 28	ND ^b^	ND	ND	ND	ND
Beef hamburger	863 ± 63	<LOD ^c^	ND	ND	ND	ND
Chicken muscle	765 ± 42	<LOD	<LOD	<LOQ ^d^	ND	ND
Turkey muscle	1000 ± 88	ND	ND	ND	ND	ND
Processed meat products						
Baked turkey muscle I	839 ± 89	<LOD	0.28 ± 0.04	ND	ND	ND
Baked turkey muscle II	1105 ± 78	0.11 ± 0.01	8 ± 1	0.32 ± 0.01	hexameta-	ND
Pre-cooked tripe	453 ± 53	2.0 ± 0.3	ND	0.9 ± 0.1	tetrameta-	ND
Cooked meat	855 ± 42	0.19 ± 0.01	<LOD	0.08 ± 0.02	ND	ND
Meat loaf	846 ± 28	<LOD	ND	ND	ND	ND
Cooked ham	931 ± 39	<LOD	3 ± 1	0.7 ± 0.2	ND	detected
Mortadella I	921 ± 88	0.67 ± 0.02	<LOD	0.3 ± 0.2	ND	ND
Mortadella II	847 ± 60	0.24 ± 0.08	ND	0.8 ± 0.2	ND	ND
Mortadella III	791 ± 24	<LOD	0.21 ± 0.07	ND	ND	ND
Mortadella IV	785 ± 69	<LOD	0.22 ± 0.03	ND	ND	ND
Wurstel I	902 ± 67	91 ± 6	11 ± 2	5 ± 1	tetrameta-, penta-	detected
Wurstel II	6094 ± 95	5 ± 2	<LOD	<LOD	ND	ND
Wurstel III	4694 ± 89	117 ± 21	ND	857 ± 99	ND	detected
Pasta with meat sauce I	647 ± 10	0.70 ± 0.03	<LOD	<LOD	ND	ND
Pasta with meat sauce II	830 ± 83	<LOD	0.21 ± 0.04	ND	ND	ND

^a^ Tetra/penta/hexapolyphosphate and their cyclic (meta) analogues for chromatographic fingerprinting with MS/MS pattern for (poly)phosphates treatment; ^b^ ND = not detected; ^c,d^ LOD = limit of detection and LOQ = limit of quantification are given in Table 2.

**Table 4:** (Poly)phosphates in different types of undeclared fish/seafood products.

	Quantification in HPIEC–HRMS					Screening in HPIEC–SCD
Sample Type	Ortho-	Pyro-	Trimeta-	Tri-	Polyphosphates HPIEC–HRMS ^a^	
	(Mean ± SD; *n* = 2; µg g^−1^)					
Fish/fish products						
Cuttlefish I	97 ± 9	<LOD ^c^	<LOD	9 ± 1	ND ^b^	ND
Cuttlefish II	944 ± 43	<LOD	<LOD	0.21 ± 0.02	ND	ND
Sea bass	987 ± 12	<LOD	<LOD	<LOQ ^d^	ND	ND
Sea bream	1159 ± 18	0.33 ± 0.07	<LOD	0.72 ± 0.03	ND	ND
Swordfish	1299 ± 29	<LOD	<LOD	1.0 ± 0.2	ND	ND
Yellowfin tuna	895 ± 40	<LOD	ND	ND	ND	ND
Salmon fillets	869 ± 86	<LOD	0.32 ± 0.09	ND	ND	ND
Tilapia fillet	652 ± 87	<LOD	<LOD	ND	ND	ND
Cod burgers	1024 ± 58	<LOD	ND	0.3 ± 0.3	ND	ND
Seafood						
Shelled mussels	141 ± 29	7 ± 1	<LOD	3 ± 1	penta-	detected
Shrimps	967 ± 15	<LOD	0.17 ± 0.02	ND	ND	ND
Squid	1187 ± 9	0.21 ± 0.04	<LOD	0.19 ± 0.07	ND	ND
Caramote prawn I	9578 ± 143	ND	9 ± 1	3 ± 1	tetra-, pentameta-penta-, hexa-	detected
Caramote prawn II	12,554 ± 179	<LOQ	ND	9 ± 1		detected
Caramote prawn III	6585 ± 29	<LOQ	5 ± 2	ND		detected

^a^ Tetra/penta/hexapolyphosphate and their cyclic (meta) analogues for chromatographic fingerprinting with MS/MS pattern for (poly)phosphates treatment; ^b^ ND = not detected; ^c,d^ LOD = limit of detection and LOQ = limit of quantification are given in Table 2.

**Table 5:** (Poly)phosphates in different types of undeclared dairy products.

	Quantification in HPIEC–HRMS					Screening in HPIEC–SCD
Sample Type	Ortho-	Pyro-	Trimeta-	Tri-	Polyphosphates HPIEC–HRMS ^a^	
	(Mean ± SD; *n* = 2; µg g^−1^)					
Milk						
Milk UHT I	681 ± 78	16 ± 2	ND ^b^	0.57 ± 0.05	ND	ND
Milk UHT II	554 ± 16	ND	<LOD ^c^	<LOD	ND	ND
Butter						
Butter I	33 ± 11	ND	ND	ND	ND	ND
Butter II	663 ± 51	ND	ND	<LOQ ^d^	ND	ND
Butter III	462 ± 88	ND	<LOD	<LOQ	ND	ND
Butter IV	786 ± 36	ND	<LOD	<LOQ	ND	ND
Cheese						
Ripened cheese	370 ± 41	ND	ND	<LOD	ND	ND
Seasoned cheese	592 ± 98	ND	<LOD	ND	ND	ND
Fresh stracchino cheese	2715 ± 184	ND	ND	49 ± 8	tetra-, tetrameta-, penta-,	ND
					hexameta-, hexa-	

^a^ Tetra/penta/hexapolyphosphate and their cyclic (meta) analogues for chromatographic fingerprinting with MS/MS pattern for (poly)phosphates treatment; ^b^ ND = not detected; ^c,d^ LOD = limit of detection and LOQ = limit of quantification are given in Table 2.
